# Expression of Concern: Visual edge feature enhancement of product appearance design images based on improved retinex algorithm

**DOI:** 10.1371/journal.pone.0360869

**Published:** 2026-10-21

**Authors:** The PLOS One Editors

After this article [1] was published, the following concerns were noted:

- The articles cited as References 1 and 22 were retracted before [1] was published.
- The specific datasets and sources of data used in this study are not described in sufficient detail to be reproducible.
- Contrary to the declaration in the Data Availability statement, the individual-level underlying data supporting the article’s results were not provided with the article, and the authors have not provided these data following editorial request. As individual-level underlying data supporting the article’s results have not been made available, this article [1] does not comply with the PLOS Data Availability policy.

In light of the concerns, the *PLOS One* Editors issue this Expression of Concern. Readers are advised to interpret the article [1] with caution.
