# Supplementary material for: Visual edge feature enhancement of product appearance design images based on improved retinex algorithm
Source: PLoS One. 2025 Sep 18;20(9):e0332195. doi: 10.1371/journal.pone.0332195 (PMC12445546; doi:10.1371/journal.pone.0332195)
Supplement: Attachment — Submitted filename: Response_auresp_2.docx [file pone.0332195.s003.docx]

Amendment notes

Journal Requirements:

If the reviewer comments include a recommendation to cite specific previously published works, please review and evaluate these publications to determine whether they are relevant and should be cited. There is no requirement to cite these works unless the editor has indicated otherwise.

Please review your reference list to ensure that it is complete and correct. If you have cited papers that have been retracted, please include the rationale for doing so in the manuscript text, or remove these references and replace them with relevant current references. Any changes to the reference list should be mentioned in the rebuttal letter that accompanies your revised manuscript. If you need to cite a retracted article, indicate the article’s retracted status in the References list and also include a citation and full reference for the retraction notice.

Additional Editor Comments:

As suggested by one reviewer, please include pseudo-code summarizing the core steps of the algorithm in the Methods section.

Re: The core algorithm steps of the visual edge feature enhancement method for the appearance design image of the above product are pseudocode, as shown in Figure 2.

[Note: HTML markup is below. Please do not edit.]

Reviewers' comments:

1. If the authors have adequately addressed your comments raised in a previous round of review and you feel that this manuscript is now acceptable for publication, you may indicate that here to bypass the “Comments to the Author” section, enter your conflict of interest statement in the “Confidential to Editor” section, and submit your "Accept" recommendation.

Reviewer #3: (No Response)

Reviewer #4: All comments have been addressed

2. Is the manuscript technically sound, and do the data support the conclusions?

The manuscript must describe a technically sound piece of scientific research with data that supports the conclusions. Experiments must have been conducted rigorously, with appropriate controls, replication, and sample sizes. The conclusions must be drawn appropriately based on the data presented.

Reviewer #3: Yes

Reviewer #4: (No Response)

3. Has the statistical analysis been performed appropriately and rigorously?

Reviewer #3: Yes

Reviewer #4: I Don't Know

4. Have the authors made all data underlying the findings in their manuscript fully available?

The PLOS Data policy requires authors to make all data underlying the findings described in their manuscript fully available without restriction, with rare exception (please refer to the Data Availability Statement in the manuscript PDF file). The data should be provided as part of the manuscript or its supporting information, or deposited to a public repository. For example, in addition to summary statistics, the data points behind means, medians and variance measures should be available. If there are restrictions on publicly sharing data—e.g. participant privacy or use of data from a third party—those must be specified.

Reviewer #3: Yes

Reviewer #4: No

Re:The statistical data in Table 1 are the mean values of PCQI, IQE, and IQM data for different methods. We have provided additional measurement data points for median and variance based on your feedback, as shown in Table 1.

5. Is the manuscript presented in an intelligible fashion and written in standard English?

PLOS ONE does not copyedit accepted manuscripts, so the language in submitted articles must be clear, correct, and unambiguous. Any typographical or grammatical errors should be corrected at revision, so please note any specific errors here.

Reviewer #3: Yes

Reviewer #4: Yes

Re: Thank you for your evaluation. I have thoroughly reviewed the paper and provided the corresponding content in response to your project feedback mentioned above.

6. Review Comments to the Author

Please use the space provided to explain your answers to the questions above. You may also include additional comments for the author, including concerns about dual publication, research ethics, or publication ethics. (Please upload your review as an attachment if it exceeds 20,000 characters)

Reviewer #3: I would like to thank the authors for submitting this outstanding paper, which presents an innovative method for enhancing image clarity and improving edge features in product appearance design using an improved Retinex algorithm. The proposed method appears effective and yields impressive results based on the metrics evaluated, such as PCQI, IQE, and IQM.

Regarding research ethics, I did not notice any concerns related to dual publication or violations of research ethics in this work. Upon reviewing the paper, it seems that all data collection and analysis were conducted transparently and in adherence to academic standards. There is no indication of this work being published elsewhere, and I believe the research makes a valuable contribution to the field of image enhancement and analysis.

I recommend the acceptance of this paper, with the continued commitment to transparency in future related publications.

Re:Thank you for your review and affirmation.

Reviewer #4: I appreciate the substantial improvements you have made in revising the manuscript. For reproducibility, please include pseudo-code summarizing the core steps of your improved algorithm in the Methods section.

Re: The core algorithm steps of the visual edge feature enhancement method for the appearance design image of the above product are pseudocode, as shown in Figure 2.

7. PLOS authors have the option to publish the peer review history of their article (what does this mean?). If published, this will include your full peer review and any attached files.

If you choose “no”, your identity will remain anonymous but your review may still be made public.

Do you want your identity to be public for this peer review? For information about this choice, including consent withdrawal, please see our Privacy Policy.

Reviewer #3: No

Reviewer #4: No
